# The earliest impact of restricted protein intake during pregnancy on heart development in male mouse offspring

**DOI:** 10.3389/fcell.2025.1678126

**Published:** 2025-10-30

**Authors:** Marina S. Folguieri, Bruno Calsa, Patricia Aline Boer, José Antonio Rocha Gontijo

**Affiliations:** Fetal Programming and Hydroelectrolyte Metabolism Laboratory, Department of Internal Medicine, Nucleus of Medicine and Experimental Surgery, FCM, Campinas, Brazil

**Keywords:** maternal protein restriction, cardiogenesis, fetal programming, apoptosis, autophagy, heart gene expression, DOHAD

## Abstract

**Introduction:**

Maternal protein restriction in animal models poses significant risks that lead to detrimental outcomes such as decreased birth weights, reduced nephron counts, neurological disorders, and increased arterial hypertension. Previous studies have shown that gestational protein deficiency is linked to reductions in cardiac mass, an increased presence of cardiac fibroblasts, and the development of fibrosis in both the left and right ventricles of adult rodents. This leads to the hypothesis that nutritional deprivation negatively impacts cellular proliferation mechanisms, ultimately resulting in a decrease in cell populations.

**Aims:**

The present study aims to elucidate the complex relationships among growth factor signaling, molecular expression profiles, and developmental pathways that underpin cardiac morphogenesis during the embryonic and fetal stages of development. We focus on the potentially harmful effects of maternal protein restriction on the cardiogenesis of the offspring.

**Methods:**

Methodologically, we analyzed the responses of female mice subjected to protein deficiency and evaluated the effects on their offspring, with a particular emphasis on early cardiac remodeling and key cellular pathways. Specifically, we analyzed cleaved caspase-3 as a marker for apoptosis, along with autophagy. Techniques employed include RT-qPCR, immunohistochemistry, and a transgenic model for direct quantification of autophagic flux.

**Results:**

Our findings indicate that gestational protein restriction alters the expression profiles of genes associated with cardiac remodeling and increases the levels of apoptotic markers. In contrast, autophagic flux did not show significant alterations.

**Conclusion:**

We conclude that early protein restriction triggers a cascade of remodeling responses, including transient heart volume expansion accompanied by cardiomyocyte hypertrophy, which may progress towards fibrosis and metabolic stress. Autophagic flux was not significantly altered. We conclude that early protein restriction triggers a cascade of remodeling responses, including a transient heart volume expansion accompanied by cardiomyocyte hypertrophy, which has the potential to progress towards fibrosis and metabolic stress.

## Graphical Abstract

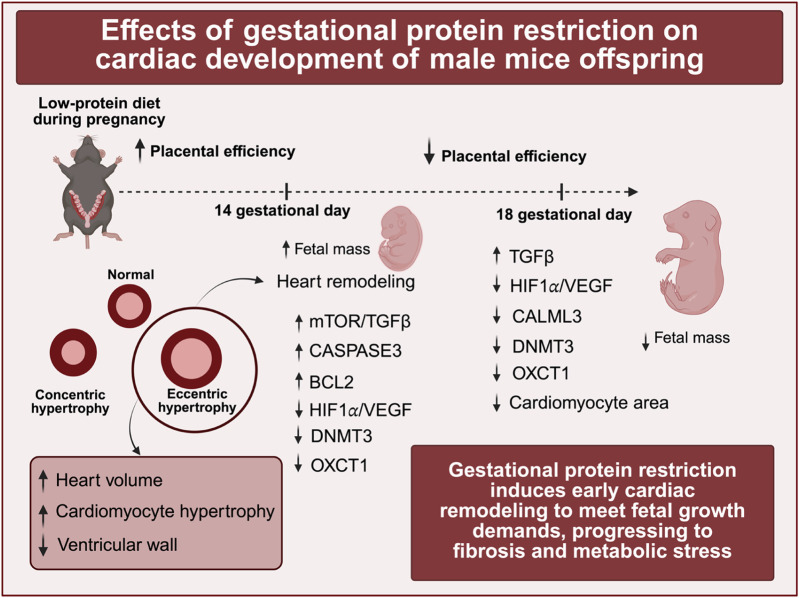

## Introduction

During the critical stages of embryonic development, the limited transport and diffusion of nutrients and oxygen through the placenta render the functioning of the developing heart essential. As one of the first organs to form and initiate its activity, the heart plays a pivotal role in supplying vital nutrients and oxygen to the fetus, thereby supporting its metabolic needs (Berhrsin and Gibson, 2011; Finnemore and Groves, 2015; Morton and Brodsky, 2016; Tan and Adam, 2020). The process of heart development commences with the migration of initial mesodermal cells, which differentiate into cardiac progenitor cells that establish two distinct molecular lineages. Both cell conception and differentiation are critical for the proper formation of the heart (Buijtendijk et al., 2020). The initial fusion of these cells forms the heart tube, which subsequently undergoes several morphological transformations, including looping morphogenesis, septation, chamber specification, and the formation of heart valves. The proliferation of cardiomyocytes is crucial for the rapid growth and development of the heart. Following birth, the cell cycle of cardiomyocyte proliferation is interrupted, and further heart growth is achieved through the hypertrophy of these cells (Li et al., 1996; Porrello et al., 2011). Complex gene regulatory networks orchestrate these developmental processes to ensure proper heart formation (Porrello et al., 2011). Disruptions that occur during early development or in the transition from intrauterine to extrauterine life can lead to long-lasting adverse effects and potentially fatal consequences (Wu et al., 2016; Cohen et al., 2016; Aye et al., 2017). According to the World Health Organization, cardiovascular disease represents the foremost cause of mortality worldwide. These diseases are frequently associated with several risk factors, including excessive alcohol consumption, tobacco use, unhealthy dietary practices, obesity, physical inactivity, and exposure to air pollution (WHO, 2024). Moreover, research conducted by Barker and colleagues has documented a higher incidence of cardiovascular diseases among individuals born with low birth weight (Barker et al., 1989a; Barker et al., 1989b). In light of Barker’s findings linking low birth weight to an increased incidence of adult diseases, a multidisciplinary research area known as the Developmental Origins of Health and Disease (DOHaD) has emerged. This field emphasizes the significant impact of early life stages—from embryonic development through early childhood—on an individual’s long-term health trajectory. Numerous studies have established correlations between developmental disorders and the onset of cardiovascular diseases in later life (Barker et al., 1989a; Barker et al., 1989b; Langley-Evans et al., 1996; Roseboo et al., 2000). Among various factors, maternal protein restriction in animal studies is noteworthy, as it has been linked to lower birth weight, reduced nephron numbers, central and peripheral neural disorders, and arterial hypertension (Mesquita et al., 2010a; Mesquita et al., 2010b; Sene et al., 2013; Custódio et al., 2017; Sene et al., 2021; Lamana et al., 2021; Folguieri et al., 2022; Mariano et al., 2021). Such maternal protein restriction has also been associated with a reduction in the number of cardiomyocytes (Corstius et al., 2005; Assalin et al., 2019) and the induction of hypertrophy in these cells (Folguieri et al., 2022; Morrison et al., 2007; Lim et al., 2006). Furthermore, gestational protein deficiency may contribute to reduced heart weight, an increased number of cardiac fibroblasts, and consequently, fibrosis in the left and right ventricles of adult rodents ultimately decreasing survival rates (Folguieri et al., 2022; Assalin et al., 2019). It is reasonable to hypothesize that a maternal protein-deficient diet may restrict the availability of essential amino acids necessary for the proliferation and differentiation of stem cells. Such limitations in protein support may alter the mechanisms and processes by which these cells multiply and differentiate. Additionally, in response to nutrient scarcity, cardiac stem cells or developing cells may undergo apoptosis (programmed cell death) or autophagy (self-digestion of cellular components), thereby reallocating crucial nutrients to support the survival and differentiation of a diminished yet viable pool of cells. Furthermore, nutrient deprivation may diminish overall proliferation processes in these organisms, resulting in a reduced cell count. The present study seeks to investigate the complex interactions among growth factors, molecular expressions, and developmental mechanisms that impact heart development during the embryonic-fetal period. Additionally, this research will emphasize the potential detrimental effects of maternal malnutrition on the heart development of offspring.

## Materials and methods

### Animals and ethical procedures

The Institutional Ethics Committee approved the experimental protocol (#5481-1/2020 CEUA/UNICAMP). The Internal Biosafety Commission (CIBIO FCM n° 08/2019) granted permission to use transgenic animals. C57BL/6J and C57BL/6-Tg (CAG-RFP/EGFP/Map1lc3b)1Hill/J (CAG-RFP-EGFP-LC3) mice, aged 8–10 weeks, were sourced from the Multidisciplinary Center for Biological Research in Laboratory Animals at Unicamp (CEMIB). The animals were housed in a controlled environment with a temperature of 23 °C ± 2 °C, a relative humidity of 50% ± 10%, and a light-dark cycle from 6:00 p.m. to 6:00 a.m. CAG-RFP-EGFP-LC3 transgenic mice (C57BL/6-Tg (CAG-RFP/EGFP/Map1lc3b)1Hill/J; RRID: IMSR_JAX:027139) express a red fluorescent protein (RFP) that remains stable at an acidic pH (pKa 4.5), alongside an enhanced green fluorescent protein (EGFP; pKa 5.9) and a gene for the microtubule-associated protein 1 light chain 3 alpha (Map1lc3a or LC3) that serves as a marker for phagosomes. The combined fluorescence of GFP and RFP produces a yellow signal in alkaline conditions in phagophores and autophagosomes. Conversely, EGFP fluorescence is quenched in autolysosomes, leading to only an RFP signal emission. Notably, 14% of EGFP in the autolysosome (pH = 4–5) continues to emit weak green fluorescence; consequently, it is essential to employ a lysosomal marker such as Lamp 1 (Novus Biologicals - NB120-19294, 1:2000) to ensure accuracy (The Jackson Laboratory, Bar Harbor, ME, United States).

### Experimental model

At 16 weeks of age, female CAG-RFP-EGFP-LC3 mice were carefully mated with male C57BL/6J mice for 2 hours during the dark cycle. Following mating, the females were transferred to individual cages and categorized into four groups based on dietary intake and gestational age for fetal collection. Some females received a standard protein diet (NP, 17% casein, n = 22), while others were administered a low-protein diet (LP, 6% casein, n = 20). Both diets were isocaloric and contained standard levels of sodium and calcium. The dams were maintained until either 14 or 18 days of gestation (GD). On the 14th or 18th gestational days (GD), the dams were anesthetized using 3% isoflurane, and the female and male fetuses were harvested promptly and weighted. Only male fetuses were utilized to eliminate potential effects of sexual programming. Some fetuses were fixed by immersion in 4% paraformaldehyde (PFA) for 4 h at 18 GD and for 3 h at 14 GD, subsequently stored in 70% alcohol. Additional fetuses were fixed in PFA for 8 h, subjected to 30% sucrose until saturation, and then frozen in N-Hexane in liquid nitrogen. After freezing, these fetuses were stored at −80 °C. Other fetuses were frozen in liquid nitrogen before being stored at −80 °C.

### Sex determination

DNA extraction was performed from tail fragments using enzymatic lysis (NaOH 50 mM) at 100 °C, and the presence of the SRY gene was assessed through conventional PCR amplification. The GoTaq® G2 Green Master Mix (M7823, Promega, Madison, United States) was employed, with the reaction consisting of 35 cycles at 95 °C, 59 °C, and 72 °C, each for 1 min. The SRY product (Forward: GTGAGAGGCACAAGTTGGC, Reverse: CTCTGTGTAGGATCTTCAATC) is 147 bp, while the positive control (MYG; Forward: TTACGTCCATCGTGGACAGC, Reverse: TGGGCTGGGTGTTAGTCTTA) yields a product of 246 bp.

### Genotyping

The investigator who provided the CAG-RFP-EGFP-LC3 did not aim to develop a homozygous colony. The Jackson Laboratory recommends mating CAG-RFP-EGFP-LC3 mice with wild-type (non-carrier - C57BL/6J). Consequently, verification of the transgene’s presence in the fetuses was essential. DNA was again extracted via enzymatic lysis (NaOH 50 mM), and genotyping was performed to confirm the presence of the transgene through conventional PCR amplification. The GoTaq® G2 Green Master Mix (M7823, Promega, Madison, United States) was utilized in a “touchdown” cycling protocol as recommended by The Jackson Laboratory. The transgene product (Forward: CAG GAC GAG CTG TAC AAG T, Reverse: CAC CGT GAT CAG GTA CAA GGA) is 208 bp, while the positive control (oIMR7338; Forward: TTACGTCCATCGTGGACAGC, oIMR7339; Reverse: TGGGCTGGGTGTTAGTCTTA) has a predicted product size of 324 bp.

### Heart volume

The cardiac volume of fixed hearts from fetuses at 14 and 18 gestational days (GD) (n = 5 per group) was calculated using the following formula: volume = π × (½ × width)^2^ × length.

### RT-qPCR

Heart tissues from fetuses at 14 GD (NP n = 3, LP n = 3) were dissected, and RNA was extracted utilizing the RNeasy Plus Micro kit (74,034—Qiagen, Hilden, Germany) according to the manufacturer’s guidelines. The NanoDrop 2000c (Thermo Fisher) was employed to evaluate the purity and concentration of the RNA, accepting samples that achieved 260/230 and 260/280 ratios of ≥1.8. For the heart from animals at 14 GD, complementary DNA (cDNA) was synthesized using 2000 ng of total RNA with the RT^2^ First Strand kit. For heart from animals at 18 GD, cDNA synthesis was conducted using the High Capacity cDNA Reverse Transcription kit. Primers were designed using the Primer-BLAST tool (NCBI) to span exon-exon junctions (see Table 1). The quantitative PCR (qPCR) cycle was performed with ReadyMix JumpStart™ Taq SYBR® (Cat. No. S4438 – Sigma Aldrich) on a StepOne Plus system under the following conditions: initial denaturation at 95 °C for 2 min, followed by 40 cycles of 95 °C for 15 s, and 60 °C (62 °C for Tgfβ at both 14 and 18 GD, and Calml3 and Myo10 at 18 GD) for 1 min. Data normalization was achieved by correcting Ct values based on the average Ct of the Gapdh.

**TABLE 1:** Primer sequence used in RT-qPCR experiment.

Gene	Sequence (5′- 3′)	Concentration
Calml3	Fwd: CAATGGCTTCGTCAGTGCAG	18DG: 62.5 nM
	Rev: CATCTGTATCTGCCGCCTGT	18 DG: 125 nM
Dnmt3a	Fwd: CCTGAGTATGAGGATGGCCG	14 GD: 300 nM and 18 GD: 150 nM
	Rev: CAGGAGAAGCCCCGAAGTTT	14 GD: 300 nM and 18 GD: 150 nM
Oxct1	Fwd: AACTACCGTGGTGGAGGTTG	14 and 18 GD: 300 nM
	Rev: TCTCCCTTTATGAGGCGGTG	14 and 18 GD: 300 nM
Tgfb1	Fwd: CGTGGAAATCAACGCTCCAC	14 and 18 GD: 75 nM
	Rev: TCCAACCCAGGTCCTTCCTA	14 and 18 GD: 75 nM
Vegfa	Fwd: TGCGGATCAAACCTCACCAA	14 and 18 GD: 300 nM
	Rev: TGTTCTGTCTTTCTTTGGTCTGC	14 and 18 GD: 300 nM
Myo10	Fwd: ACAACGGACAGGTGGACAAG	18 GD: 75 nM
	Rev: TGACGTGGCAGCTAGCTTTT	18 GD: 75 nM
Hif	Fwd: GGCGAGAACGAGAAGAAAAATAGG	14 and 18 GD: 500 nM
	Rev: -ACTCTTTGCTTCGCCGAGAT	14 and 18 GD: 500 nM
Mtor	Fwd: GCATAACAGATCCTGACCCTGAT	14 and 18 GD: 300 nM
	Rev: TTCAGAGCCACAAACAGAGC	14 and 18 GD: 300 nM
Gapdh	Fwd: ACCCTTAAGAGGGATGCTGC	14 and 18 GD: 300 nM
	Rev: CCCAATACGGCCAAATCCGT	14 and 18 GD: 300 nM

### Immunohistochemistry

Fetuses preserved in 70% alcohol underwent dehydration, clearing, and embedding in Paraplast (Sigma-Aldrich, United States). Five-micrometer-thick tissue sections were deparaffinized and prepared for both immunoperoxidase and immunofluorescence assays. The slides were treated with PBS (pH 7.2) for hydration and washing for 5 min. Antigen retrieval was conducted in a water bath using citrate buffer (pH 6.0) for 30 min. The slides were incubated with a blocking solution of 5% non-immune serum from the secondary antibody’s host species for 1 h for the immunoperoxidase method. Following re-washing with PBS, the sections were incubated overnight at 4 °C with a primary antibody diluted in 3% BSA. Endogenous peroxidase activity was subsequently inhibited with hydrogen peroxide, and the slides were treated with methanol for 10 min in the dark. After additional PBS washes, the sections were exposed to a specific secondary antibody, diluted in 1% BSA, for 2 h at room temperature and visualized using DAB (3.3′-diaminobenzidine tetrahydrochloride, Sigma-Aldrich CO®, United States). After a final wash with running water, the slides were dehydrated and mounted with a coverslip using Entellan®. For immunofluorescence, the slides were incubated with a blocking solution (containing 5% non-immune serum from the secondary antibody’s host species and 0.2% Triton in PBS). The heart sections were incubated with the primary antibody diluted in this blocking solution overnight at 4 °C. After washing with PBS, the sections were treated with the specific secondary antibody diluted in the blocking solution for 2 h and 30 min (Table 2). During the final PBS wash, DAPI (0.1 μg/mL, D9564-10 MG–Sigma) was added. Immunofluorescence images were obtained using an Airyscan Inverted Microscope at the National Institute of Science and Technology on Photonics Applied to Cell Biology (INFABIC) at the State University of Campinas. Images for the immunoperoxidase method were captured using an Axio Scope A1 microscope equipped with an Axio Cam MRc digital camera (Carl Zeiss, Oberkochen, Germany). No immunoreactivity was observed in negative control experiments in which one of the primary antibodies was excluded. Image analysis was performed utilizing ImageJ software.

**TABLE 2:** Antibodies used with their appropriate concentrations and supplies.

Antibody	Dilution	Antibody supplies
LAMP 1	1:2000	NovusBio - NB120-192994
PCNA	1:500	Santa Cruz- SC25280
CLEAVED CASPASE-3	1:600	Cell Signaling- 9662S
mTOR	1:300	Sigma- SAB4300583
AMPKα	1:300	Santa Cruz- SC7446
Anti-Mouse - Alexa 488 linked	1:200	Biomol- A21202
Anti- Rabbit - Dylight 594 linked	1:200	Abcam- AB9840
Anti-Rabbit IgG Alexa 647 linked	1:2000	Jackson Imuno Resrarch- 711-605-152
HIF1α	1:200	Novus- NB11057031
VEGF	1:200	Novus- NB100-664
BCL2	1:100	Santa Cruz- SC492
CALML3	1:2000	NovusBio- NBP2-15667
DNMT3a	1:200	Bioss - BS0497R
OXCT1	1:1500	Bioss - BS5089R
Anti-Rabbit IgG HRP linked	1:200	Cell Signaling- 7074S
Anti-Mouse IgG HRP linked	1:200	Invitrogen- 31,430

### Autophagic flux

**FIGURE 1:**
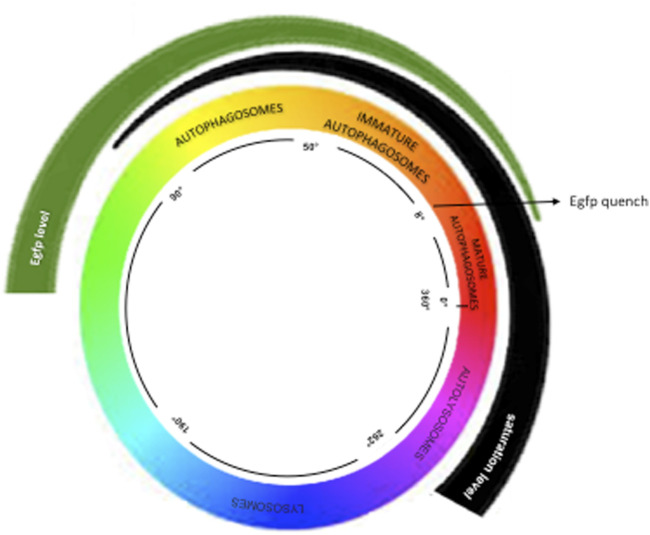
Hue Method—This method categorizes colors by angles, as described in https://www.mrexcel.com/forum/excel-questions/559852-rgb-hue-formula.html. Vesicles between 50° and 80° are yellow; autophagosomes between 9° and 50° are orange; immature autolysosomes (0°–8° and 356°–360°) are red; fused autolysosomes (263°–357°) are purple; lysosomes (190°–262°) are blue.

Images of the hearts (n = 4 per group) were obtained using a confocal microscope with Airyscan (Carl Zeiss), which includes the following laser lines: 1. DAPI: 404 nm 2. eGFP (green): 488 nm 3. mRFP (red): 543 nm 4. Alexa 647 (blue): 633 nm. The images were captured using an EC Plan-Neofluar 40x/1.3 Oil DIC objective, accompanied by a 1.8x zoom. Automated selection of autophagic vesicles was executed in ImageJ software using the following command line: run (“8-bit”); run (“Gaussian Blur”, “sigma = 1″); run (“Subtract Background”, “rolling = 30″); run (“Auto Threshold,” “method = Default”); setOption (“BlackBackground,” false); run (“Convert to Mask”); run (“Watershed”); run (“Particle Remover” “size = 40-Infinity pixel show = Masks summarize overlay add”); The mean intensity of each vesicle was measured in the respective red, green, and blue channels (RGB), facilitating classification of vesicle color based on the HUE angle. The classification of vesicle colors, as delineated by Lee et al. (2019), included the following categories: Yellow vesicles (HUE 51–80), Orange (9–50), Red (0–8, 357–360), Purple (263–356), and Blue (190–262). For the total count of autophagic vesicles in the heart, those classified as yellow, orange, red, and purple were considered based on area (number of vesicles per mm^2^) (see Figure 1).

### Data presentation and statistical analysis

All data were reported as mean ± standard deviation of the mean (SD). Student's *t*-tests were used to evaluate studies involving only two independent samples, within or between groups. Welch’s test was used to correct situations characterized by heteroscedasticity (different variances between groups). Statistical analyses for gene expression data were performed on the raw ΔΔCT values, with fold change values calculated as 2^−ΔΔCT^. GraphPad Prism 5.00 was used for data analysis (GraphPad et al., United States). The level of significance was set at p ≤ 0.05.

## Results

### Maternal parameters

At 14 days of gestation, dams in the control group (NP) demonstrated a greater weight gain (9.230 ± 0.8984 g; n = 11) in comparison to the low-protein group (LP), which exhibited a weight gain of 6.970 ± 0.7582 g (n = 9), with a statistically significant difference (p < 0.0001). Feed consumption was similar between the groups, with NP consuming 47.19 ± 2.728 g (n = 10) and LP consuming 48.63 ± 2.514 g (n = 9), resulting in a p-value of 0.125. However, feed efficiency was significantly diminished in the LP group (0.03690 ± 0.004894; n = 5) compared to the NP group (0.04894 ± 0.005461; n = 5), yielding a p-value of 0.0063. At 18 days of gestation, weight gain remained higher in the NP group (16.15 ± 1.680 g; n = 11) relative to the LP group (10.89 ± 1.423 g; n = 11), with p < 0.0001. Notably, feed consumption was greater among LP dams (67.13 ± 6.432 g; n = 11) than among NP dams (59.07 ± 8.575 g; n = 12), with a p-value of 0.0195. Nonetheless, feed efficiency remained lower in the LP group (0.04417 ± 0.01199; n = 5) compared to the NP group (0.06697 ± 0.01680; n = 5), with a p-value of 0.0387.

### Fetal parameters

At 14 days of gestation, male fetuses in the LP group demonstrated a significantly higher body mass (0.211 ± 0.02 g; n = 40) than those in the NP group (0.199 ± 0.02 g; n = 46; p = 0.005). The placental mass did not exhibit a significant difference between the two groups (NP: 0.103 ± 0.03 g; n = 46; LP: 0.099 ± 0.02 g; n = 41; p = 0.5). The fetal-to-placental (f:p) ratio for male fetuses did not reveal a significant difference between the LP (2.286 ± 0.66; n = 40) and the NP (2.074 ± 0.67; n = 46) groups (p = 0.07). Among female fetuses, body mass was also higher in the LP group (0.206 ± 0.02 g; n = 35) in comparison to the NP group (0.189 ± 0.02 g; n = 41; p = 0.001), while placental mass remained comparable (NP: 0.097 ± 0.03 g; n = 40 vs. LP: 0.099 ± 0.03 g; n = 35; p = 0.7). The female f:p ratio exhibited no significant difference between the groups (NP: 2.049 ± 0.62; n = 41 vs. LP: 2.163 ± 0.59; n = 33; p = 0.2). At 18 days of gestation, male fetal body mass was slightly lower in the LP group (0.939 ± 0.09 g; n = 39) compared to the NP group (0.979 ± 0.085 g; n = 49; p = 0.03), while placental mass remained consistent between groups (NP: 0.115 ± 0.03 g; n = 49; LP: 0.116 ± 0.02 g; n = 39; p = 0.9). The f:p ratio in male fetuses did not show significant differences (NP: 8.983 ± 2.3; n = 39 vs. LP: 8.411 ± 1.772; n = 39; p = 0.2). Among female fetuses, body mass was significantly lower in the LP group (0.895 ± 0.08 g; n = 44) compared to the NP group (0.977 ± 0.107 g; n = 27; p = 0.0006). There was a non-significant reduction in placental mass in the LP group (0.099 ± 0.03 g; n = 44) when compared to NP (0.111 ± 0.03 g; n = 27; p = 0.09). The f:p ratio remained statistically similar between the groups (NP: 8.917 ± 2.1; n = 26 vs. LP: 9.52 ± 2.2; n = 44; p = 0.2).

### Heart development

**FIGURE 2:**
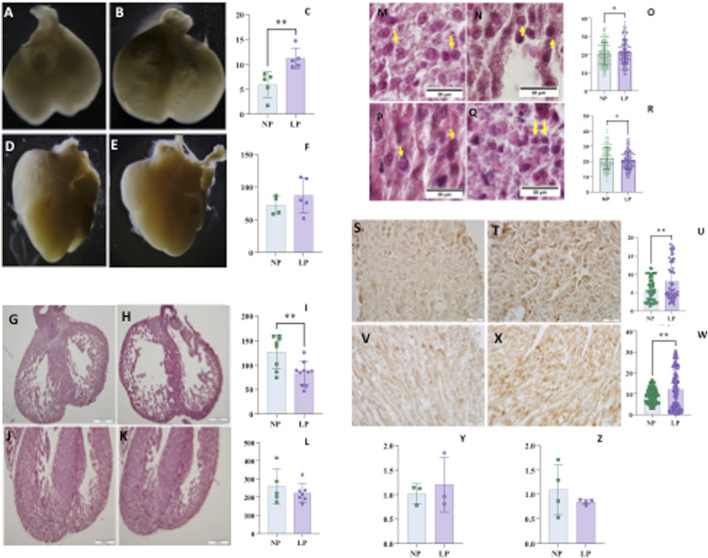
Heart volume normalized by body mass of fetuses at 14 days of gestation (panel **C**), NP: 28,26 ± 12,26 n = 5 vs. LP: 54,14 ± 8,102 n = 5, p = 0,0043, and NP illustrated picture (Panel **A**) and LP illustrated picture (Panel **B**). At 18 days of gestation (panel **F**), NP: 73,83 ± 21,15 n = 4 vs. LP: 84,29 ± 28,04 n = 4, p = 0,5728, and NP illustrated picture (panel **D**) and LP illustrated picture (panel **E**). Ventricle wall thickness at 14 GD (panel **I**), NP: 126,9±34,30 n = 8 vs. LP: 83,03±23,55 n = 10, p = 0,0053, and NP illustrated picture (panel **G**) and LP illustrated picture (panel **H**). At 18 gestational days (panel **L**), NP: 259642±96285 n = 5 vs. LP: 223422±50656 n = 7, p = 0.4132, and NP illustrated picture (panel **J**) and LP illustrated picture (panel **K**). Myocyte area at 14 GD (panel **O**), NP: 20,56±6,216 n = 352 vs. LP: 21,55±6,462 n = 306, p = 0,047, and NP illustrated picture (panel **M**) and LP illustrated picture (panel **N**). At 18 gestational days (panel **R**), NP: 22,24±7,248 n = 243 vs. LP: 20,85±5,866 n = 268, p = 0,0171, and NP illustrated picture (panel **P**) and LP illustrated picture (panel **Q**). TGFΒ1 in heart with 14 GD (panel **U**), NP: 5,892±3,111 n = 54 vs. LP: 8,152±5,215 n = 57 and p = 0,0069 and, NP illustrated picture (panel **S**) and LP illustrated picture (panel **T**); At 18 GD (panel **W**) NP: 9,227±3,464 n = 109 vs. LP: 12,09 ± 9,269 n = 108 and p = 0,0028 and, NP illustrated picture (panel **V**) and LP illustrated picture (panel **X**). Tgfb1 gene expression in heart at 14 GD (panel **Y**) to NP: 1,015 ± 0,2021 n = 3 compared to LP: 1,204 ± 0,5594 n = 3 and p = 0,3052. While the Tgfb1 gene expression in heart at 18 GD to NP: 1,096 ± 0,5118 n = 4 (panel **Z**) compared to LP: 0,8471 ± 0,05234 n = 4 and p = 0,3705. For gene expression data, statistical analyses were performed on the raw ΔΔCT values.

For the assessment of cardiac parameters, only male offspring were analyzed. At 14 days of gestation, the cardiac volume was greater, while the ventricular wall thickness was reduced in the LP group. By 18 days of gestation, no significant alterations were observed in cardiac volume or left ventricular thickness; however, myocyte area was smaller in the LP group compared to the control group at this gestational age, whereas it was increased at 14 days (Figure 2).

### Gene expression

A significant difference in mTOR expression was observed at 14 days of gestation (14 GD), where an increase in mRNA levels was noted in the low-protein (LP) group. No significant differences were detected in the expression of other mRNAs, namely Oxc1, Dnmt3a, Vegfa, Hif1a, Tgfb1, Calml3, Myo10, at 18 GD, nor in mTOR expression at this gestational age (refer to Figures 5, 6).

### Autophagy process

**FIGURE 3:**
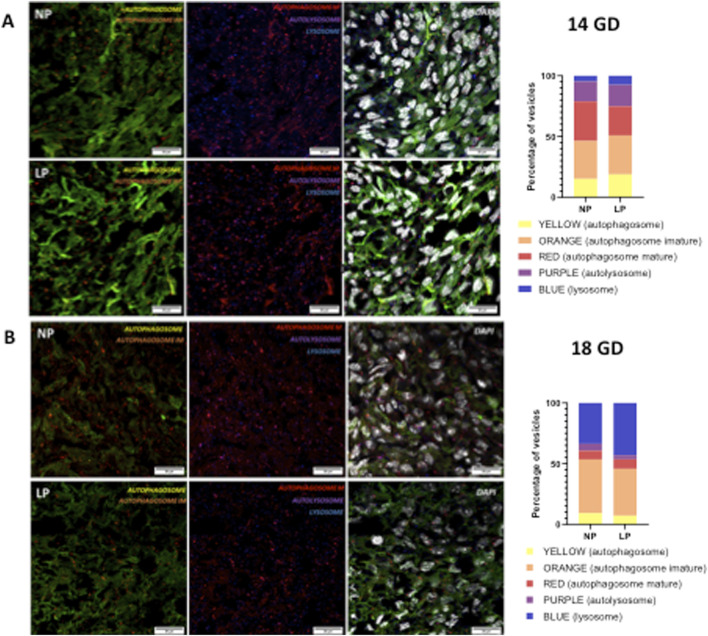
Illustrates the autophagy process at 14 gestational days (GD) (panels **(A)**) and at 18 GD (panels **(B)**). Both the NP and the LP offspring groups are shown. The combination of GFP (green fluorescent protein) and RFP (red fluorescent protein) fluorescence produces a yellow signal in phagophores and autophagosomes. In immature autophagosomes, EGFP is quenched, resulting in an orange signal, while mature autophagosomes emit only the RFP signal. Upon fusion with lysosomes, the autolysosome produces a purple signal (indicating RFP + LAMP1). No differences were observed in any of the autophagy vesicles between the two gestational ages.

The progression of the autophagy process in the myocardial tissue of subjects subjected to gestational protein restriction did not demonstrate any significant changes at either 14 GD or 18 GD (as illustrated in Figure 3).

### Apoptosis and proliferation

**FIGURE 4:**
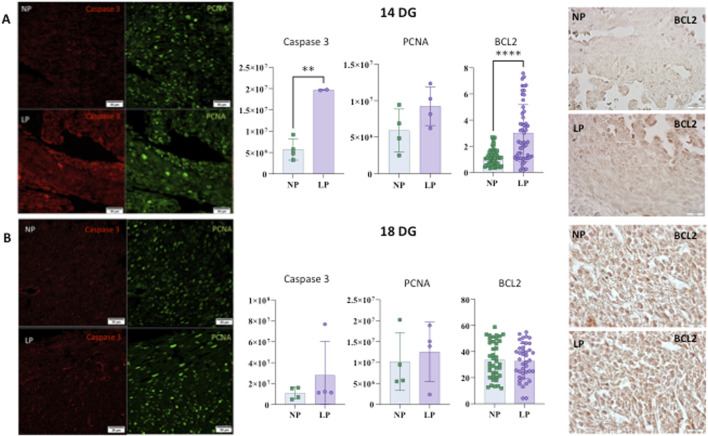
Illustrates the levels of Caspase, PCNA, and BCL2 in the heart at 14 gestational days (GD) (panel **(A)**). For Caspase-3: NP: 5,753,514 ± 2,511,538 (n = 4) and LP: 19,673,098 ± 121,515 (n = 3), p = 0.0018. For PCNA: NP: 5,899,701 ± 2,943,273 (n = 4) and LP: 9,228,202 ± 2,652,340 (n = 4), p = 0.1439. For BCL2: NP: 1.119 ± 0.6136 (N = 59) and LP: 3.014 ± 2.172 (N = 56), p < 0.0001. (panel **(B)**) shows the levels of Caspase, PCNA, and BCL2 in the heart at 18 gestational days (GD). For Caspase-3: NP: 10,932,348 ± 5,676,129 (n = 4) and LP: 28,005,548 ± 32,553,962 (n = 3), p = 0.3413. For PCNA: NP: 10,214,501 ± 6,904,684 (n = 4) and LP: 12,527,971 ± 7,116,435 (n = 4), p = 0.6572. For BCL2: NP: 33.86 ± 14.17 (n = 40) and LP: 32.75 ± 13.55 (n = 40) - p = 0.7228.

At 14 GD, an increase in activated caspase 3, a key executor of apoptosis, was observed. However, no differences were identified regarding the cell proliferation marker, PCNA, at either 14 GD or 18 GD. Additionally, no changes were observed in activated caspase three levels at 18 GD (see Figure 4).

### Autophagy controllers

At 14 GD, mTOR levels were elevated; however, no significant differences were found at 18 GD, nor were any differences noted in AMPKα at either gestational age (refer toFigure 5).

**FIGURE 5:**
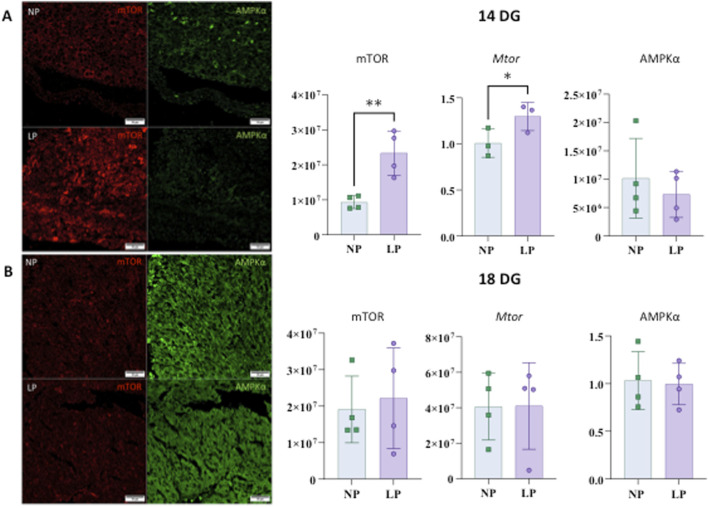
mTOR and AMPKα heart immunostaining at 14 gestational days (panels **(A)**). The results for mTOR are as follows: NP: 9,323,377 ± 1,848,170 n = 4, and LP: 23,337,483 ± 6,352,528 n = 4, p = 0.0055. For gene expression of Mtor: NP: 1,008 ± 0.1532 n = 3 and LP: 1,297 ± 0.1524 n = 3, p = 0.0407. The results for AMPKα are as follows: AMPKα: NP: 10,162,846 ± 7,037,267 n = 4 and LP: 7,332,373 ± 4,066,103 n = 4, p = 0.5122. Immunostaining to mTOR and AMPKα in the heart at 18 GD (panels **(B)**). mTOR: NP: 19,110,748 ± 9,139,961 n = 4 and LP: 22,152,485 ± 13,815,193 n = 4, p = 0.7260. Gene expression of Mtor shows NP: 1,031 ± 0.3035 n = 4 and LP: 0.9956 ± 0.2176 n = 4, p = 0.8547. For AMPKα: NP: 40630609 ± 18834851 n = 4 and LP: 41,010,226 ± 24,429,236 n = 4, p = 0.9812.

### Cell death regulator

An increase in BCL-2 expression, which is part of a gene family comprising both pro-apoptotic and anti-apoptotic members, was observed at 14 GD. Conversely, no significant differences in BCL-2 expression were detected at 18 GD (see Figure 4).

### Hypoxia regulator

**FIGURE 6:**
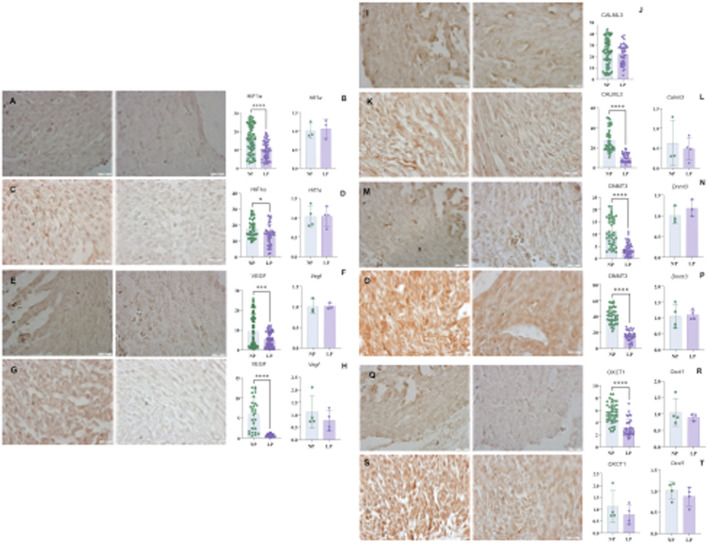
HIF1α immunohistochemistry in heart with 14 GD (panel **(A)**): NP: 15.80 ± 7.56, n = 90 and LP 9,978 ± 4,878, n = 73, p < 0.0001. And Hif1α gene expression (panel **(B)**) NP: 1,014 ± 0.2106, n = 3, and LP: 1,070 ± 0.2629, n = 3, p = 0.7872. For HIF1α in the Heart (panel **(C)**) at 18 GD: NP: 17.57 ± 5,834 n = 38, and LP: 13.88 ± 6,405 n = 39, p = 0.0101. And Hif1α gene expression (panel **(D)**) NP: 1,025 ± 0.2669 n = 4 and LP: 1,035 ± 0.2444 n = 4 and p = 0.9562. To VEGF (panel **(E)**) heart at 14 GD (panel **(E)**): NP: 9,489 ± 8,069, n = 91, LP- 5,381 ± 3,767, n = 70, p = 0.0001. And, to Vegf gene expression (panel **(F)**) NP: 1,010 ± 0.1799 n = 3 and LP- 1,020 ± 0.07310, n-3, p = 0.9306. For VEGF in the Heart at 18 GD **(G)**: NP: 6,088 ± 4,021, n = 28, LP: 0.6854 ± 0.3730, n = 27, p < 0.0001. And, Vegf gene expression **(H)**: NP: 1,113 ± 0.6625, n = 4, LP: 0.7750 ± 0.4086, n = 4, p = 0.4185. For CALML3 in Heart at 14 GD (panels **(I,J)**): NP: 23.54 ± 11.89 n = 72 and LP: 22.20 ± 8,627 n = 46, p = 0.5098. And heart CALML3 at 18 GD **(K)**NP- 27.64 ± 11.48, n = 40, LP: 10.49 ± 4,526 n = 30, p < 0.0001. For Calml3 gene expression (panel **(L)**), NP: 0.6275 ± 0.5688 n = 3 and LP: 0.4835 ± 0.2845 n = 4, p = 0.6736. DNMT3 immunohistochemistry in Heart at 14 GD **(M)**: NP: 11.13 ± 5,794, n = 44 and LP-3,988 ± 22,615, n = 41, p < 0.0001. And Dnmt3a gene expression (panel **(N)**) to NP: 1,014 ± 0.2114 n = 3 and LP: 1,184 ± 0.2076, n = 3, p = 0.3760. Immunohistochemistry DNMT3 in Heart with 18 GD NP (panel **(O)**): 40.87 ± 9,965, n = 36, LP: 15.46 ± 6,535 n = 32, p < 0.0001. (panel **(P)**) shows Dnmt3a gene expression to NP: 1,049 ± 0.3745, n = 4 and LP - 1,099 ± 0.1523, n = 4, p = 0.8126. For OXCT1 immunohistochemistry in the Heart with 14 GD (panel **(Q)**) NP: 5,474 ± 1,586, n = 45 and LP– 3,175 ± 1,420, n = 31, p < 0.0001. And Oxct1 gene expression (panel **(R)**) NP: 1,053 ± 0.4190, n = 4 and LP: 0.8953 ± 0.1082 n = 3, p = 0.5612. And OXCT1 (panel **(S)**) in the Heart with 18 GD: NP: 34.22 ± 20.29 n = 40 and LP: 15.51 ± 12.46, n = 40, p < 0.0001. And, (panel **(T)**) shows Oxct1 gene expression: NP: 1,016 ± 0.1975 n = 4 and LP: 0.8762 ± 0.2221, n = 4, p = 0.3831.

Hypoxia-inducible factors (HIF-1 alpha) exhibited a decrease in the myocardial tissues of subjects subjected to gestational protein restriction at both 14 GD and 18 GD. Furthermore, the vascular endothelial growth factor (VEGF), which HIF-1α upregulates, was also found to be decreased at both gestational ages (illustrated in Figure 6, panels A–H).

### Calcium signaling

In the LP group, CALM3 levels were reduced by 18 GD when compared to the normal protein (NP) group. However, no differences in CALM3 levels were observed at 14 GD (see Figure 6, panels I–L).

### DNA methylation

A decrease in DNA methyltransferase 3 (DNMT3) levels was observed at both gestational ages, 14 GD and 18 GD (as shown in Figure 6, panels M–P).

### Heart development metabolism marker

The levels of 3-oxoacid CoA-transferase 1 (OXCT1) were decreased at both 14 GD and 18 GD in the LP group in comparison with the control group (refer to Figures 6Q–T).

### Transforming growth factor beta 1 (TGFβ1)

The level of TGFβ1 was found to be elevated in both 14 GD and 18 GD in the LP group when compared to the control group (illustrated in Figures 2U,W).

## Discussion

In the present study, mothers assigned to the low-protein (LP) group showed increased food consumption at 18 days of gestation. Nonetheless, this increase did not yield sufficient caloric efficiency, resulting in inadequate weight gain compared to dams in the normal protein intake (NP) group. Similar observations were noted at 14 days of gestation, further supporting the conclusion that mothers on a low-protein (LP) diet experienced diminished weight gain. These findings are congruent with the existing literature, which indicates that gestational protein restriction is associated with reduced weight gain during pregnancy (Desai et al., 1996; Passos et al., 2000; Colombo et al., 1992; Toyomizu et al., 1991; França et al., 2009). Moreover, gestational protein restriction is frequently linked to intrauterine growth restriction and low birth weight (Mesquita et al., 2010a; Mesquita et al., 2010b; Sene et al., 2013; Lamana et al., 2021; Desai et al., 1996). However, our current research offers a novel perspective, illustrating that gestational protein restriction initially results in an increase in body weight, heart volume, and myocyte area in male mice at 14 days of gestation. Nevertheless, by 18 days of gestation, the male mouse offspring subjected to gestational protein restriction exhibit reduced body weights. The current data suggest that, in some instances, limiting maternal protein intake can result in an initial increase in fetal body weight during the early stages of gestation. However, as the pregnancy progresses, this trend frequently reverses, resulting in decreased fetal body weight among protein-restricted groups compared to control groups (Langley-Evans et al., 1996; L et al., 1998; Rees et al., 1999). Such alterations may affect the initial increase in fetal body mass within the placenta, influenced by the secretion of both fetal and maternal hormones. Although the phenotypic plasticity of the placenta may support fetal growth to some extent, its efficiency tends to decline as gestation advances, rendering the fetus more susceptible to the detrimental effects of a low-protein diet, which results in a diminished supply of essential amino acids (Fowden et al., 2009). This study exclusively examined placental mass and did not assess additional placental parameters. The increase in heart volume during development represents a complex and highly regulated process that encompasses various structural and functional adaptations essential for meeting the growing organism’s requirements. This growth primarily relies on an increase in the number of cardiomyocytes, resulting from vigorous cellular proliferation. Hypertrophy also occurs, particularly during intermediate and later developmental stages, in response to hemodynamic and metabolic demands. These processes work together, allowing the heart to expand and adapt functionally to the needs of the developing organism (Li et al., 1996; Porrello et al., 2011; Crispi et al., 2020). The increment in heart volume observed in the low-protein (LP) group of animals at 14 days of gestation may be attributed to hypertrophy, despite no corresponding increase in PCNA expression. The type of hypertrophy present in the heart is intricately linked to the hemodynamic conditions and physiological processes experienced during growth. The heart’s adaptive response can yield two distinct forms of hypertrophy: eccentric hypertrophy and concentric hypertrophy. Concentric hypertrophy occurs when the ventricular wall thickens, directly correlated with the increased load on the ventricle. This form of hypertrophy is more prevalent in scenarios of pressure overload, where the ventricle must exert greater effort to pump blood against increased pressure. Eccentric hypertrophy is predominantly observed during the initial stages of growth and is a physiological response to volume overload. This overload is characterized by an increase in circulating blood volume, particularly as a fetus develops and its metabolic demands escalate. The heart compensates for this elevated volume by undergoing enlargement. In the context of eccentric hypertrophy, ventricular dilation occurs to a greater extent than the thickening of the ventricular walls. This results in a significant increase in the size of the ventricular cavity, but fails to achieve a proportional increase in wall thickness. Furthermore, volume overload can stimulate hypertrophy in cardiomyocytes, subsequently enhancing the heart’s ability to generate force (Mihl et al., 2008; Eghbali et al., 2005). The augmentation of both heart volume and cardiomyocyte area, without a corresponding thickening of the ventricular wall, may facilitate an enhancement in circulating blood volume, thereby supporting fetal growth by approximately 14 days of gestation. Given that the heart is the first organ to develop and attain functionality (Berhrsin and Gibson, 2011; Finnemore and Groves, 2015; Morton and Brodsky, 2016; Tan and Adam, 2020), it is plausible that it completes its proliferative phase earlier than other organs, subsequently adapting through a process of remodeling. Hypertrophy serves as a compensatory mechanism that enables surrounding organs to adjust to their physiological requirements. Initially, cardiomyocyte hypertrophy empowers the heart to generate increased force, thereby meeting heightened demands for blood flow (Li et al., 1996; Porrello et al., 2011; Crispi et al., 2020). However, sustained volume overload can precipitate pathological changes, including fibrosis and compromised heart function. Transforming Growth Factor-beta (TGF-β) plays a crucial role in this transition. While it initially promotes cellular growth, excessive levels can lead to increased extracellular matrix deposition and fibrosis (Dobaczewski et al., 2011; Frangogiannis, 2017). Moreover, elevated levels of the mammalian target of rapamycin (mTOR) may further amplify the effects of TGF-β on fibrotic processes. This enhancement may occur through direct modulation of TGF-β signaling or by creating conditions conducive to the activation of fibrotic pathways, such as augmented oxidative stress or the activation of pro-inflammatory factors (Jiménez et al., 2021; Shin et al., 2025; Saadat et al., 2021; Igarashi et al., 2021; Li et al., 2023). Consequently, heart volume in the offspring LP group may resemble that of the control group, despite a loss of cellular integrity associated with heightened Caspase-3 activity resulting from extracellular matrix deposition and fibrosis. The selection of markers such as Caspase-3, PCNA, and mTOR is grounded in their established significance within the literature as indicators of specific cellular pathways: apoptosis, proliferation, and nutrient signaling, respectively. One of the primary functions of the mTOR pathway is to inhibit autophagy, a cellular process that can lead to programmed cell death. Notably, mTOR also modulates apoptosis independently of autophagy, thereby regulating cellular demise through both autophagy-dependent and autophagy-independent mechanisms. Empirical research has shown that mTOR can simultaneously inhibit autophagy while promoting apoptosis; however, the precise mechanisms underlying this dual effect remain only partially understood. Consequently, it is reasonable to conclude that mTOR exhibits bidirectional regulation of cell death (Xie et al., 2023; Xu et al., 2020; Wang and Wenchao, 2021). Furthermore, mTOR activates caspase-3 to induce apoptosis via the IRE/JNK signaling pathway (Kato et al., 2012). Bcl-2 is recognized as an anti-apoptotic protein that protects cells from programmed cell death and plays an integral role in development (Czabotar et al., 2014). Similar to AMPK, Bcl-2 is involved in regulating growth and is associated with pro-autophagy processes (Mihaylova and Shaw, 2011). Data collected on the 14th day of gestation revealed an increase in caspase-3, a marker for cell death, and independent of autophagy, suggesting an elevation in cell death. Nevertheless, in the present study, the concurrent increase in Bcl-2 indicates that the developing heart, under conditions of gestational protein restriction, is in a precarious balance between cell survival and apoptosis. This finding emphasizes the complexity of cellular responses to nutritional stress during pregnancy. Moreover, Bcl-2 may form a complex with Beclin 1 (Decuypere et al., 2012), which has the potential to inhibit autophagy. Such inhibition may impede the clearance of damaged cellular structures, thereby promoting cell death. In the current study, a decrease in the levels of DNA methyltransferase 3 (DNMT3) was observed on gestational days 14 and 18. Previous research suggests that maternal protein restriction can lead to epigenetic modifications, including alterations in DNA methylation patterns. Between embryonic days 11.5 and 14.5, the DNA methylation profile in the developing mouse heart remains predominantly stable, with changes detected in only a limited subset of genes linked to cardiac development. Additionally, undifferentiated cardiomyocytes, or primary cardiac myocytes derived from embryonic stem cells, exhibit elevated levels of methylation compared to the embryonic stem cells from which they are derived. This observation underscores the crucial role of DNA methylation in controlling the expression of genes that encode cardiac structural proteins. Notably, the knockdown of DNA (cytosine-5)-methyltransferase 3A (Dnmt3a) in mouse embryonic cardiomyocytes leads to disrupted sarcomere assembly, reduced beating frequency, and impaired contractile function (Lan and Evans, 2019; Chamberlain et al., 2014). The Calml3 protein functions as an intracellular calcium sensor and is critical for the expression and stability of Myosin 10 (Rogers and Strehler, 2001; Bennett et al., 2007; Bennett et al., 2008).

A reduction in Calml3 expression is associated with decreased levels of Myosin 10, which may contribute to cardiac abnormalities, including impaired calcium signaling, reduced myocyte contractile capacity, and abnormal cardiac morphology. Mice exhibiting a 20% reduction in Myosin 10 expression demonstrate significant cardiac functional impairments by the 14th embryonic day, including decreased fractional shortening, lower heart rate, and increased systolic diameter of the left ventricle (Ma and Adelstein, 2014). Consequently, reduced CALM3 levels in our model may contribute to long-term defects in excitation–contraction coupling and cardiac remodeling. Hypertrophy of the heart is characterized by a reduction in the number of cardiomyocytes at birth, accompanied by progressive myocyte hypertrophy, altered nuclear morphology, interstitial fibrosis, infiltration of inflammatory cells, an increase in the levels of cleaved CASPASE-3, a key marker of the cardiomyocyte apoptotic pathway, and compromised cardiac contractile function (Ma et al., 2009). Accordingly, the observed decline in Calml3 protein levels in fetuses at 18 gestational days subject to intrauterine protein restriction may be correlated with hypertrophy, diminished populations of diverse cardiomyocyte types, interstitial fibrosis, and other modifications noted in the hearts of animals affected by gestational protein restriction (Silva et al., 2013; Folguieri et al., 2022; Assalin et al., 2019). This phenomenon may lead to morphological changes in the left ventricle, comprising a decrease in the number of myocytes and binucleated myocytes, as well as an increase in fibrosis and apoptosis, as illustrated by studies utilizing this experimental model (Assalin et al., 2019). Throughout embryonic development, tissues frequently encounter low oxygen levels, or hypoxia. The hypoxia-inducible factor 1-alpha (HIF-1α) acts as a pivotal transcription factor activated under such conditions. HIF-1α facilitates the expression of adaptive genes that enhance cellular survival, regulates the proliferation and differentiation of cardiomyocytes and other cell types, and contributes to cardiac formation and the development of new blood vessels by directly activating the transcription of vascular endothelial growth factor (VEGF) (Knutson et al., 2021; Guimarães-Camboa et al., 2015; Compernolle et al., 2003; Ryan et al., 1998; Kotch et al., 1999). The decline in HIF-1α and its target gene, VEGFA, observed in animals from the low-protein (LP) group may be associated with inhibited cell proliferation and heightened cell death (Gomes et al., 2023). Such responses are adaptive mechanisms to ensure nutrient availability in a constrained environment, permitting only a limited number of viable cells to develop and thrive. Nonetheless, after an increase in nutrient availability post-birth, the alterations that initially supported development may predispose individuals to disease, as reported in various studies addressing the consequences of gestational protein restriction (Folguieri et al., 2022; Corstius et al., 2005; Morrison et al., 2007; Lim et al., 2006). Oxct1 (3-oxoacid CoA-transferase 1) is the rate-limiting enzyme in ketone body oxidation, enabling the heart to utilize ketones as an efficient energy substrate, particularly under stress conditions such as ischemia or nutrient deprivation (Cotter et al., 2013; Maalouf et al., 2007; Veech, 2004). Reduced OXCT1 expression during fetal development may therefore compromise the ability of cardiomyocytes to adapt metabolically, rendering them more vulnerable to energy deficits and oxidative stress later in life. Together, the reduction in OXCT1 and CALM3 observed in protein-restricted fetuses highlights an early metabolic and structural vulnerability that may predispose the heart to maladaptive remodeling and cardiovascular disease in later life. Therefore, the diminished expression of Oxct1 and HIF-1α observed in the low-protein (LP) group at gestational day 14 indicates that these animals are already exhibiting signs of metabolic stress that persist until day 18 of gestation. In conclusion, while placental adaptations may appear advantageous for specific organs and facilitate their development, the ramifications of protein restriction become increasingly pronounced in the later stages of gestation. In contrast, the heart undergoes remodeling earlier in the developmental timeline. Thus, these findings indicate that early gestational protein restriction significantly alters the expression profiles of genes involved in heart development, influencing cell proliferation and autophagy dynamics. This suggests a cascade of early cardiac remodeling responses aimed at accommodating fetal growth requirements. Notably, this process may lead to transient heart volume expansion accompanied by cardiomyocyte hypertrophy, which has the potential to progress towards fibrosis, metabolic stress, and a reduction in ventricular wall thickness. We must include a statement on study limitations, acknowledging that our findings lay a strong groundwork for future large-scale investigations. However, subsequent transcriptomics (RNA-Seq) or proteomics studies will yield a more comprehensive perspective and represent logical, necessary steps.

## Data Availability

The datasets presented in this study can be found in online repositories. The names of the repository/repositories and accession number(s) can be found in the article/supplementary material.
